# From a Somatotopic to a Spatiotopic Frame of Reference for the Localization of Nociceptive Stimuli

**DOI:** 10.1371/journal.pone.0137120

**Published:** 2015-08-28

**Authors:** Annick L. De Paepe, Geert Crombez, Valéry Legrain

**Affiliations:** 1 Department of Experimental-Clinical and Health Psychology, Ghent University, Ghent, Belgium; 2 Institute of Neuroscience, Université catholique de Louvain, Brussels Woluwe, Belgium; University of Muenster, GERMANY

## Abstract

To react efficiently to potentially threatening stimuli, we have to be able to localize these stimuli in space. In daily life we are constantly moving so that our limbs can be positioned at the opposite side of space. Therefore, a somatotopic frame of reference is insufficient to localize nociceptive stimuli. Here we investigated whether nociceptive stimuli are mapped into a spatiotopic frame of reference, and more specifically a peripersonal frame of reference, which takes into account the position of the body limbs in external space, as well as the occurrence of external objects presented near the body. Two temporal order judgment (TOJ) experiments were conducted, during which participants had to decide which of two nociceptive stimuli, one applied to either hand, had been presented first while their hands were either uncrossed or crossed over the body midline. The occurrence of the nociceptive stimuli was cued by uninformative visual cues. We found that the visual cues prioritized the perception of nociceptive stimuli applied to the hand laying in the cued side of space, irrespective of posture. Moreover, the influence of the cues was smaller when they were presented far in front of participants’ hands as compared to when they were presented in close proximity. Finally, participants’ temporal sensitivity was reduced by changing posture. These findings are compatible with the existence of a peripersonal frame of reference for the localization of nociceptive stimuli. This allows for the construction of a stable representation of our body and the space closely surrounding our body, enabling a quick and efficient reaction to potential physical threats.

## Introduction

To react efficiently to stimuli that affect the integrity of the body, we have to localize them precisely. Thanks to a good spatial acuity, the nociceptive system seems finely-tuned for the localization of noxious stimuli on the body surface [1, 2]. However, the localization of noxious stimuli requires not only the identification of their position on the body, but also the identification of their position in external space [3]. Information from the body surface and information from the external world are believed to be integrated in peripersonal frames of reference, which code both the position of somatosensory stimuli on the body surface *and* the position of stimuli in external space (e.g. visual stimuli) *if* presented in close proximity to the body. This idea has been investigated for touch (see [4]). Regarding nociception, we suggested the existence of such a peripersonal frame of reference for the localization of nociceptive stimuli [5]. In that study participants had to perform temporal order judgments (TOJs) on pairs of nociceptive target stimuli, one applied to either hand at various stimulus onset asynchronies (SOAs). Participants had to decide which hand was stimulated first. Slightly before the presentation of the first nociceptive stimulus, a visual stimulus was presented either in close proximity of one of the hands, or far from the hands (i.e. 70 cm in front if the hands). It was found that the visual stimulus speeded the perception of the nociceptive stimulus applied to the ipsilateral hand, at the detriment of the nociceptive stimulus applied to the opposite hand. More importantly, this effect was stronger when the visual stimulus was presented near the participants' hands, as compared to trials in which it was presented far away. These results suggest that the processing of nociceptive stimuli is affected by the occurrence of visual stimuli located in the peripersonal space of the body. Based upon these findings, we suggested that nociceptive stimuli can be mapped according a peripersonal frame of reference.

In the present study we wanted to confirm this hypothesis by showing that the spatial perception of nociceptive stimuli is made through a remapping of the body space according a spatial frame of reference which takes into account the relative position of the body limbs in external space. Indeed, when hands are in normal posture (as was the case in the study of De Paepe et al. [5]), the somatosensory and the visual maps are merely aligned, in the sense that the visual and the nociceptive inputs are sent to the same hemisphere. Therefore, our previous results were not able to completely dissociate between effects resulting from crossmodal displacement of spatial attention on the somatotopic representation of the skin surface from effects resulting from a remapping of nociceptive processing according to external space coordinates (i.e. a spatiotopic frame of reference) (see [6]). Such spatiotopic frame of reference allows taking into account the relative positions of body parts in external space, enabling us to recognize that when the left hand is displaced toward the right side of space, objects approaching the right space are now approaching the left hand instead of the right hand. Here, we would like to demonstrate that the positions of nociceptive stimuli can be completely remapped according a spatial representation of the body. To this end we used a crossing hand procedure, that is, when the relative position of the hands in external space is manipulated according to the sagittal midline of the body. Indeed, crossing the hands over the body midline generates a mismatch between the somatotopic and spatiotopic representations, enabling to dissociate between these two types of reference frames. This procedure makes it then possible to test whether the ability to perceive the spatial position of a somatosensory stimulus on the body is only based on the hemispheric projection of the somatosensory receptive field, or also on the relative position of the stimulated limb in external space.

For tactile information, such dissociation has been shown in studies with patients with right hemisphere lesions. For example, Smania and Aglioti [7] showed that the ability of patients with hemispatial neglect and/or tactile extinction to detect somatosensory stimuli applied to the left hand changed according to the location of the hand in external space. Whereas the perception of stimuli applied to the left hand was poor in an uncrossed posture, especially when the right hand was concurrently stimulated, the perception was improved when the left hand was crossed over the body midline and was positioned in the right side of space. These results demonstrate that the somatosensory deficits of these patients are not only linked to the anatomical projection of sensory inputs to a damaged hemisphere, but also to a defective computation of body-centered spatial coordinates. Moreover, they showed that the coding of the spatial position of the hands depends on their relative positions in external space, irrespective to their positions from the body midline [8].

In healthy volunteers the existence of a spatiotopic reference frame has been demonstrated using tactile TOJ tasks and crossmodal congruency tasks. Studies using the TOJ task have frequently found that participants could correctly report the temporal order of two tactile stimuli when the hands were uncrossed, but often misreport the order when the hands were crossed over the body midline [9–12]. In these tasks, participants were probably confused due to a competition between a somatotopic reference frame and a remapping of the tactile stimuli according to spatiotopic coordinates [9]. In the crossmodal congruency task with tactile targets and visual distractors, it was shown that the interference of visual stimuli on tactile processing was space-based. In the crossed posture the discrimination of tactile stimuli applied to the left hand was more influenced by right- than left-sided visual stimuli, and vice versa [13–17]. This result was not observed in a split-brain patient showing that remapping somatosensory information according to space-based reference frames is not possible when the cortical hemispheres are disconnected [18].

In monkeys, the ability to remap tactile inputs according to a peripersonal frame of reference has been suggested to rely on the existence of bimodal visuotactile neurons that have been reported in the ventral premotor cortex and the ventral intraparietal sulcus of the monkey [19]. Bimodal cells are cells that fire both for tactile stimuli and for visual stimuli, presented near the stimulated area. For instance, Graziano, Hu, and Gross [20] showed that the visual receptive fields (RFs) of these bimodal cells are remapped when the monkey’s posture changes, i.e., the visual RFs follow the hands in space as different postures are adapted.

For nociceptive stimuli, it has been shown that crossing the hands over the body midline affects the judgments concerning the temporal order of nociceptive stimuli applied to either hand [21], and even the perception of their intensity [22]. The fact that crossing the hands affects the temporal sensitivity of participants suggests that nociceptive processing is influenced by the conflict generated by the crossing hands procedure between the somatotopic representation of the body, and a spatiotopic representation. These studies demonstrate the usefulness of the crossing hands procedure to investigate the remapping of nociceptive stimuli applied to the body in a space-based frame of reference.

In the present study we used the crossing hands procedure and investigated the contribution of posture to code the position of nociceptive stimuli applied to a specific body part relative to external stimuli occurring close to that body part. This was investigated in two TOJ experiments, during which participants had to decide which of two nociceptive stimuli, one applied to either hand at various SOAs, had been perceived to occur first while their hands were either in an uncrossed or a crossed posture. The occurrence of the nociceptive stimuli was cued by visual stimuli. In Experiment 1, these cues were presented both in near and far space. In Experiment 2, the cues were only presented in near space. We hypothesized that, if the spatial coding of nociceptive stimuli is accounted only by the hemispheric projection of the sensory inputs, visual information on the left side of space would always prioritize stimuli presented to the left side of the body, and vice versa. The ability to report the perception of a nociceptive stimulus applied to one hand should not be affected by crossing the hands. Conversely, if nociceptive stimuli are mapped in a spatiotopic frame of reference, visual information in the left side of space would prioritize nociceptive stimuli presented to the left hand when hands are uncrossed, but to the right hand when hands are crossed (and vice versa for visual stimuli in the right side of space). The closer the visual stimulus to the body, the stronger should be this bias. In addition, the participants should be less accurate in reporting the temporal order of the nociceptive stimuli when the hands are crossed.

## Methods

### 2.1. Experiment 1

#### 2.1.1. Participants

Twenty-two paid participants volunteered to take part in this experiment. One participant was excluded because of the use of antidepressant medication at the time of the experiment. The mean age of the 21 remaining participants (17 women; 19 right-handed) was 23 years (ranging from 19 to 38 years). All of the participants had normal to corrected-to-normal vision. History of neurological, psychiatric or chronic pain diseases, and usual intake of psychotropic drugs were considered as exclusion criteria. The experimental procedure was approved by the ethics committee of the faculty of psychology and educational sciences of the UGent (2014/46). All of the participants provided written informed consent prior to taking part in the study.

#### 2.1.2. Stimuli and apparatus

The nociceptive stimuli were delivered by means of intra-epidermal electrical stimulation (IES) (DS7 Stimulator, Digitimer Ltd, UK), with stainless steel concentric bipolar electrodes (Nihon Kohden, Japan; [23]). The electrodes consisted of a needle cathode (length: 0.1 mm, Ø: 0.2 mm) surrounded by a cylindrical anode (Ø: 1.4 mm). By gently pressing the device against the participant’s skin, the needle electrode was inserted into the epidermis of the dorsum of the hand in the sensory territory of the superficial branch of the radial nerve. Using intra-epidermal stimulation at maximum twice the absolute threshold was shown to selectively activate the free nerve endings of the Aδ fibers [23–25]. The detection threshold was determined with single-pulse stimuli (0.5 ms square wave pulse) using a staircase procedure [26]. The detection threshold was established separately for each hand. Next, the stimulus intensity was set at twice the detection threshold. If necessary, the intensity of the stimuli was adjusted so that the stimuli delivered to each hand were perceived as being equally intense. During the course of the experiment proper, the stimuli consisted of trains of three consecutive 0.5 ms square-wave pulses separated by a 5-ms inter-pulse interval. Using a set of pain words from the Dutch McGill Pain questionnaire [27] the stimuli were described as pricking. After each experimental block, the participants were asked to estimate the intensity elicited by the nociceptive stimuli on a numerical graphic rating scale (10 cm) with the following labels selected from the Dutch version of the McGill pain questionnaire [26] (0 = felt nothing, 2.5 = lightly intense, 5 = moderately intense, 7.5 = very intense, 10 = enormously intense). This scale was used in order to ensure that: (1) the stimuli were still perceived, and (2) the percept elicited by the IES delivered to each of the participant’s hands was still equivalent. If one of these two criteria was not met, the stimulus intensities were modified accordingly (with a maximum increase of 0.10 mA). If this adaptation proved to be unsuccessful (i.e. one of the criteria was still not met), the electrodes were displaced and the procedure was restarted.

The visual stimuli were presented by means of four green light-emitting diodes (LEDs). The LEDs were illuminated for 20 ms, and these stimuli were perceived by participants as a green light that briefly flashed. In a practice phase, the visibility of each of the LEDs was tested by asking the participants to report on the location of the LED that was illuminated (e.g., ‘left near’, ‘right far’).

#### 2.1.3. Procedure

**Fig 1:**
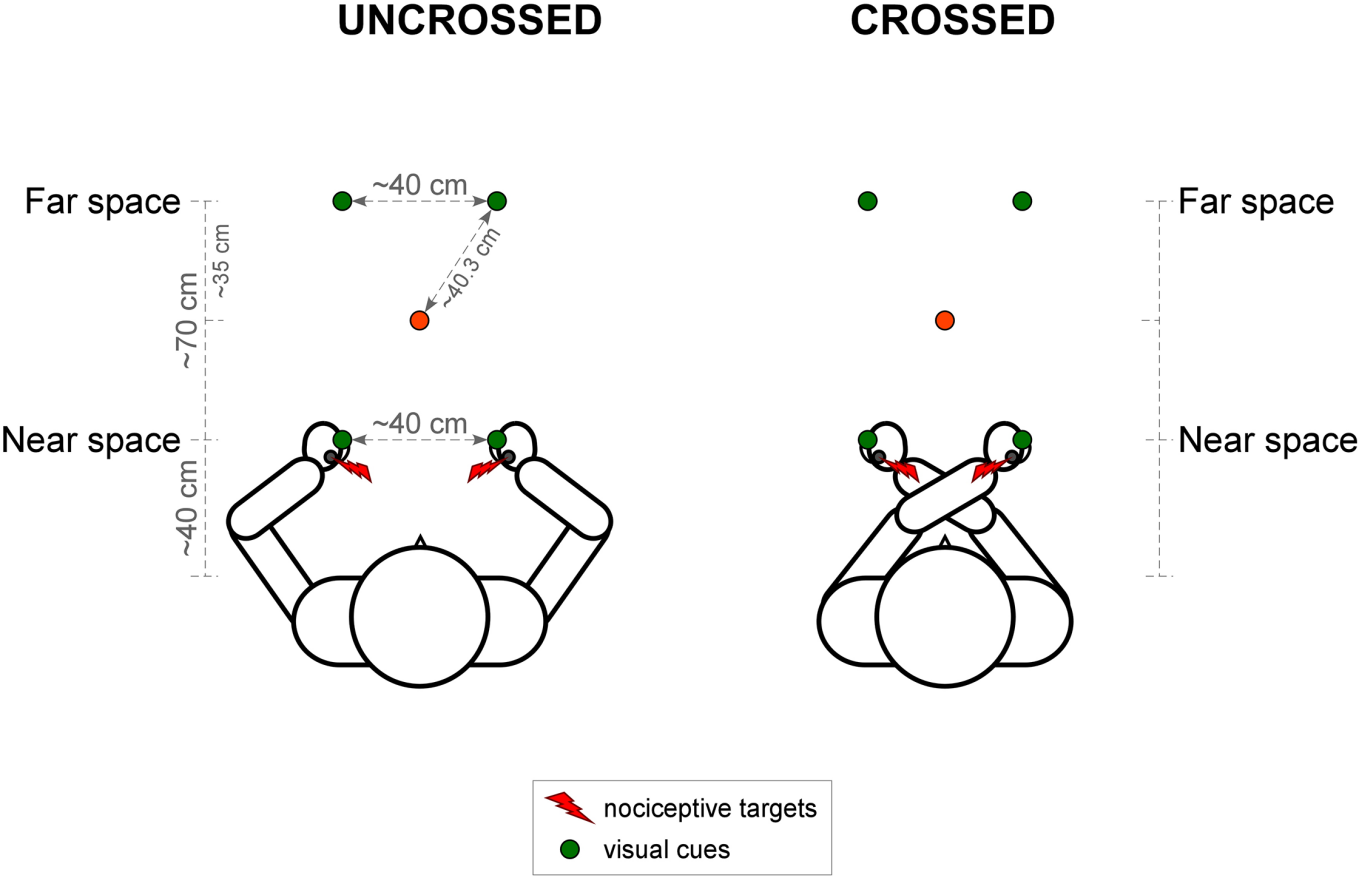
Illustration of the experimental set-up for Experiment 1. Nociceptive stimuli, represented by the red lightning symbols, were applied to both of the participant’s hands. Visual cues, represented by the green circles, were presented at four different locations in each trial: either unilaterally or bilaterally, either on the participant’s hands (in near space) or in front of the participant’s hands (in far space). Participants were fixating on a red LED (represented by the red circle) that was positioned in between the LEDs in near and far space. In half of the blocks participants were asked to cross their hands over the body midline (right side of the figure). In Experiment 2, the set-up was identical, except for the fact that the LEDs were only presented in near space.

The participants sat on a chair in a dimly illuminated, sound-attenuated room. They rested their arms on the table in front of them. The distance between the participant’s hands and their trunk, as well as the distance between the participant’s index fingers was 40 cm. The participant’s head was immobilized in a chin-rest positioned at 10 cm from the trunk, in order to prevent movements of the head. The height of the chin-rest was individually adapted. Two of the LED’s were placed in near space, and two in far space. The LEDs in near space were positioned 40 cm from the trunk, in between thumb and middle finger. The distance between the two LEDs was approximately 40 cm. The LEDs in far space were situated 70 cm in front of the LEDs in near space. Participants were fixating on a red LED positioned equidistantly from the LEDs in far and near space, and equidistantly from the left and right LEDs (see Fig 1). Responses were given by means of two foot pedals, one positioned under the toes, and one under the heel. Participants were instructed to keep the foot pedals depressed, and to either raise their heel or their toes very briefly to respond which hand was stimulated first. Half of the participants responded with their left foot, the other half with their right foot. The response mapping (toe = left hand, heel = right hand, or vice versa) was counterbalanced between participants. Participants were instructed to be as accurate as possible, speed was not important.

To get used to the stimulus response mapping, a first practice session contained 1 block of 20 trials, in which participants were presented with one IES target, either on the left or the right hand. Participants indicated, by means of the foot pedals, which hand was stimulated. In a second practice phase of 2 blocks (one with the uncrossed and one with the crossed posture) of 36 trials participants practiced the actual experiment with cues and nociceptive targets, but only using the three largest SOAs, to ensure correct task performance. The experiment did not proceed until participants had 80% correct performance on the largest SOAs in both blocks.

The actual experiment consisted of 8 blocks of 60 trials. Four blocks contained visual stimuli in near space only, and four blocks visual stimuli in far space only. The order of the blocks was randomized for the first 4 blocks and the reversed order was used for the last 4 blocks. In half of the blocks participants were asked to cross their hands, one arm over the other. The posture (crossed/uncrossed) of the arms was alternated over blocks and the order was counterbalanced. In half of the crossed hands blocks, participants had to cross their left arm over their right arm. In the other half they had to cross their right arm over their left arm. The order was again counterbalanced.

A trial started with the fixation LED being illuminated. This fixation LED stayed on during the entire trial. 500 ms after the onset of the fixation LED, the visual stimulus was presented either in near or far space. The visual stimulus consisted of either a single unilateral flash occurring in left space, a single unilateral flash occurring in right space, or two flashes resulting from the bilateral and simultaneous illumination of the LEDs on both sides. The visual stimulus was followed 80 ms later by a pair of nociceptive stimuli, one applied to either hand. The first nociceptive stimulus could be applied either to the left or the right hand. Five possible SOAs were used between the two nociceptive stimuli for each order of stimulation (left hand first vs. right hand first): ±200, ±90, ±55, ±30, ±10 ms (where positive values indicate that the participant’s right hand was stimulated first, and negative values indicate that their left hand was stimulated first).

The trials were created combining 3 spatial locations of the visual stimuli (unilateral left, unilateral right or bilateral) x 2 orders for the nociceptive stimuli x 5 SOAs. Trials were randomly presented within each block of stimulation. The visual cues were spatially uninformative, and the location of any forthcoming nociceptive stimulus could thus not be predicted by the cue.

Participants were instructed to keep their gaze on the fixation LED throughout each block of trials and to indicate by means of the foot pedals, which hand was stimulated first, irrespective of the side of space in which their hand was located. After the participants had made their response, the fixation LED was turned off. If participants did not respond within 10s, the fixation LED flickered 3 times before the experiment continued. After 1000 ms, the next trial started. The experiment took on average 75 minutes.

#### 2.1.4. Measures

**Fig 2:**
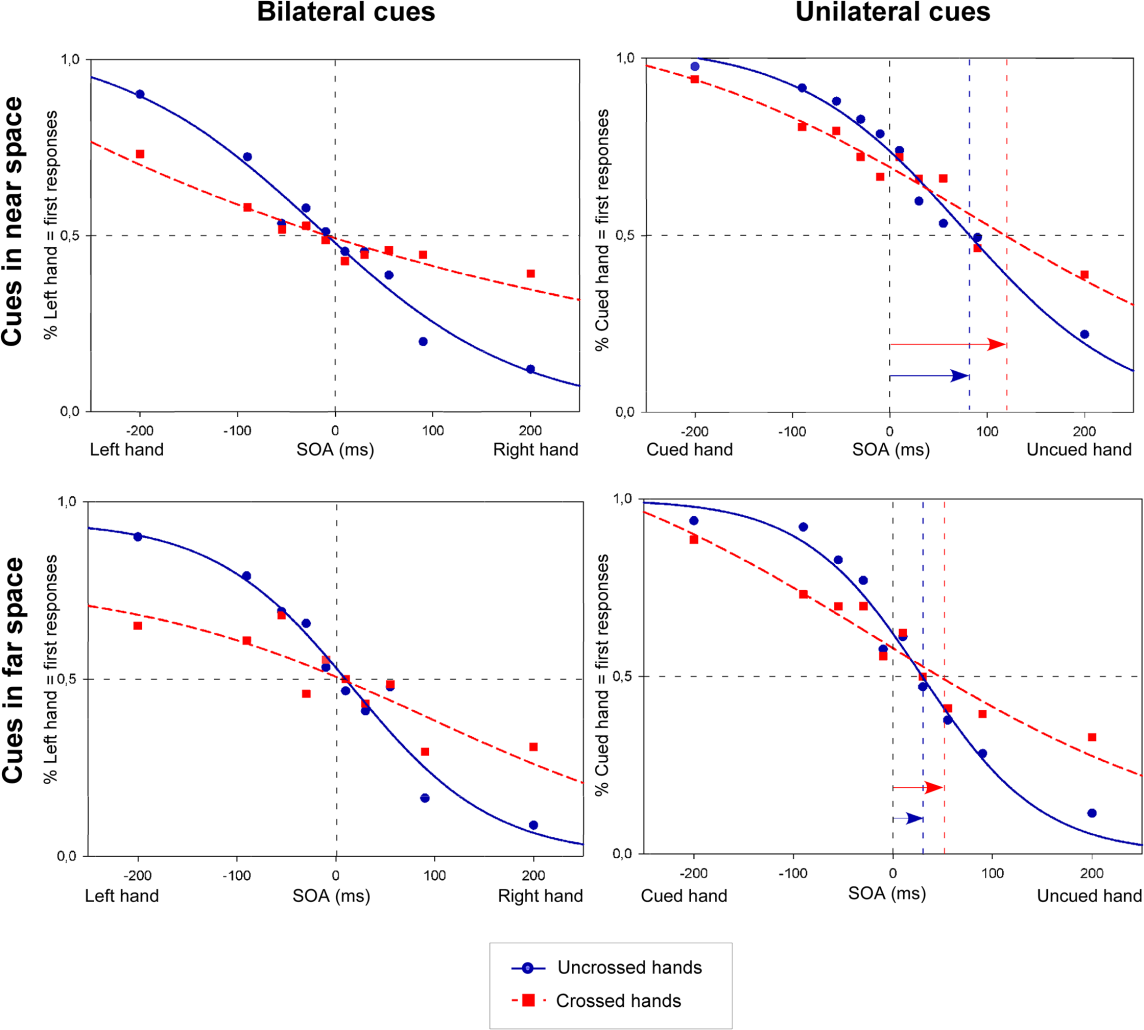
Nociceptive temporal order judgments (TOJs) in Experiment 1. The figures illustrate the fitted curves from cumulative data from 21 participants. Trials were either associated with bilateral cues (left side of the figure), or with an unilateral cue (right side of the figure), with cues in near space (upper part figure), or with cues in far space (lower part figure), and with an uncrossed (blue solid line) or a crossed (red dashed line) posture. Data are plotted as the mean proportion of *left hand first responses* (on the Y-axis; left side of figure, bilateral cues) or *cued hand first responses* (on the Y-axis; right side of figure, unilateral cues), as a function of the stimulus onset asynchronies (SOA) (on the X-axis). On the X-axis, for the bilateral cue conditions, the negative values of the SOAs indicate that the left hand was stimulated first, and the positive values indicate that the right hand was stimulated first. In the unilateral cue condition, negative values indicate that the cued hand was stimulated first, while positive values indicate that the uncued hand was stimulated first. The blue (uncrossed hands) and red (crossed hands) vertical dashed lines, and the length of the blue and red arrow coincide with the PSS values. Compared to the bilateral cue conditions, the curves in the unilateral cue conditions were shifted toward the uncued side both for the uncrossed and the crossed posture. This indicates that the nociceptive stimulus on the uncued hand had to be presented several milliseconds before the stimulus on the cued hand to have an equal chance to be perceived first, regardless of posture. The JND characterizes the slope of the functions: the steeper the slope, the lower the JND and the higher the temporal sensitivity (and vice versa).

The procedure followed that reported in Spence et al. [28] (see also [29, 30]). For each participant, and for each SOA for each of the 8 within-participant conditions (bilateral vs. unilateral cues x near vs. far space x uncrossed vs. crossed), the proportion of trials on which participants perceived the cued hand as being stimulated first, was calculated. A sigmoid function was fitted to these proportions (see Fig 2). Subsequently, the proportion of *left/right hand first responses* (left hand when the cue was presented at the side of space in which the left hand was situated, and right hand first when the cue was presented at the side of space in which the right hand was situated) was converted into *z*-scores by means of a standardized cumulative normal distribution (probits). The best-fitting straight line was computed for each participant and each condition, and the derived slope and intercept values were used to compute the point of subjective simultaneity (PSS) and the just noticeable difference (JND). The PSS refers to the point at which participants report the two events (i.e., the nociceptive stimuli presented to the right and left hand) as occurring first equally often. This is equivalent to the SOA value corresponding to a proportion of *left/right hand first responses* of 0.5 [28]. The PSS is computed as the opposite of the intercept divided by the slope from the best-fitting straight line. In the unilateral cue condition, the sign of the PSS for the conditions in which the cues were presented on the side of space where the right hand was positioned, was reversed and for each participant the final PSS value was calculated by taking the average of the PSS values for cues presented at the position of the left hand, and the reversed PSS value for cues presented at the position of the right hand. Hence, the PSS reflects how much time the stimulus at the *uncued hand* had to be presented before/after the *cued hand* in order to be perceived as having occurred at the same time. In the bilateral cue condition, there was no “cued” or “uncued” hand, as cues were always presented bilaterally. We decided to calculate the PSS from the amount of *left hand first* responses. The PSS for the bilateral cue trials thus reflects how much time the stimulus at the *right hand* has to be presented before/after the *left hand* stimulus in order to be perceived as presented at the same time. In sum, the PSS provides information concerning biases in spatial attention resulting from the presentation of the visual cues. In order to control whether the side at which the visual cue was presented could have influenced the PSS values in unilateral cue trials, we did a separate analyses including *Side of the visual stimulation* as a factor. These analyses showed that merging PSS values for cues presented on the left and the right side of space will not distort the results (see data in S1 Appendix).

The JND was measured as 0.675/slope [28]. This corresponds to the value achieved by subtracting the SOA at which the best fitted line crosses the 0.75 point from the SOA at which the same line crosses the 0.25 point, and dividing this by two) and indicates the interval needed to achieve 75% correct performance, and, as such provides a standardized measure of the sensitivity of participant’s temporal perception.

#### 2.1.5. Analyses

Analyses were performed on the PSS and JND values. PSS values that exceeded twice the maximum SOA were excluded from the data, together with their corresponding JND values. Extremely large PSS values indicate that participants were not able to perform the task correctly even at large SOA’s, where the task performance is expected to be nearly perfect. Therefore, the results in some conditions are missing for some participants. In order to test if this was influenced by the position of the hands, the difference in missing values between the uncrossed and the crossed posture condition was compared using a chi-squared test for equality of proportions.

To address the question of whether there was an attentional bias (due to the capture of attention by the visual cues), we tested whether the PSS differed significantly from 0, using one-sample t-tests.

Next, in order to compare the PSS across the different conditions, results were analyzed using the linear mixed effects models as implemented in the R package “Linear and Nonlinear Mixed Effects Models” [31]. Linear mixed effects models account for the correlations in within-subjects data by estimating subject-specific deviations (or random effects) from each population-level factor (or fixed factor) of interest (see [32] for an elaboration). We chose to analyze the data with linear mixed models because it is a more subject-specific model and it allows unbalanced data, unlike the classical general linear models which requires a completely balanced array of data [32].

The primary outcome variable was the Point of Subjective Simultaneity (PSS). The independent variables were the *Laterality* (unilateral/bilateral), the *Cue Distance* (near/far) and the *Posture* (uncrossed/crossed). These were manipulated within subjects. Each analysis required three steps. First, all relevant factors and interactions were entered in the model as fixed factors, and we assessed whether it was necessary to add a random effect for each of the fixed factors in the analysis: If a random effect significantly increased the fit of the model, it was included in the final model. By default, a random effect was added introducing adjustments to the intercept conditional on the *Subject* variable. In the second step, we searched for the most parsimonious model that fitted the data. To achieve this, we systematically restricted the full model, comparing the goodness of fit using likelihood-ratio tests. Finally, in the third step, we inspected the ANOVA table of the final model and tested specific hypotheses about possible main effects or interactions (for a similar approach see [33–36]). Kenward-Roger approximations to the degrees of freedom were used to adjust for small sample sizes [37]. When an interaction effect was significant, it was further investigated with follow-up contrast analyses, corrected for multiple testing according to the Holm-Bonferroni corrections [38]. Standardized regression coefficients were reported as a measure of the effect size. The models are presented in the supporting information (Tables A-C in S1 File).

The same method was used to assess the influence of the different experimental conditions on the JND. The models are presented in the supporting information (Tables G-I in S1 File).

### 2.2. Experiment 2

#### 2.2.1. Participants

Seventeen paid participants volunteered to take part in this experiment. The mean age of the participants (12 women; 12 right-handed) was 19 years (ranging from 18 to 22 years). All of the participants had normal to corrected-to-normal vision. History of neurological, psychiatric or chronic pain diseases, and usual intake of psychotropic drugs were considered as exclusion criteria. The experimental procedure was approved by the ethics committee of the faculty of psychology and educational sciences of the UGent (2014/46). All of the participants provided written informed consent prior to taking part in the study.

#### 2.2.2. Stimuli and apparatus

The experimental set-up was largely similar to the set-up of Experiment 1. As we were mostly interested in the effect of the posture (uncrossed/crossed) on the interaction between nociceptive and visual inputs in peripersonal space, the LEDs in Experiment 2 were only presented in near space. The distance between the participants’ hands and their trunk, as well as the distance between their index fingers was 40 cm. The two LEDs were presented in between thumb and index finger. The same procedure was used to determine the detection threshold.

In order to reduce the number of rejected values from the dataset compared to Experiment 1, we used a larger range of SOA’s between the two nociceptive targets: ±600, ±400, ±250, ±100, ±70, ±50, ±30, ±15 (positive values indicate that the right hand was stimulated first, negative values indicate that the left hand was stimulated first). Due to technical failure of the foot pedals, responses were given verbally.

#### 2.2.3. Procedure

The practice session contained 2 blocks (one uncrossed, one crossed) of 18 trials with nociceptive targets only with the three largest SOAs to ensure correct task performance (80% correct performance was required in both conditions (uncrossed/crossed) for the maximum SOA), and 2 blocks (one uncrossed, one crossed) of 18 trials with the cues and the targets (again only the three largest SOAs were used and 80% correct performance was required in order to proceed with the experiment). The experiment consisted of 4 blocks of 96 trials. In two blocks participants were asked to cross their hands, in the other two blocks hands were uncrossed. The order was alternated and counterbalanced across participants. In half of the crossed hands blocks, participants had to cross their left arm over their right arm, in the other half they had to cross their right arm over their left arm. The order was again counterbalanced.

A trial started with the fixation cross being illuminated. This fixation LED stayed on during the entire trial. 500 ms after the onset of the fixation LED, a single unilateral visual flash (either on the right or left side), or paired bilateral visual flashes were presented. The visual stimulus was followed 80 ms later by a pair of nociceptive stimuli, one applied to either hand. Eight possible SOAs were used between the two nociceptive stimuli for each order of stimulation (left hand first vs. right hand first): ±600, ±400, ±250, ±100, ±70, ±50, ±30, ±15 ms (positive values indicate that the right hand was stimulated first, negative values indicate that the left hand was stimulated first). Each block of trials was created by combining the 3 spatial locations of the visual stimuli (unilateral left, unilateral right or bilateral) x 2 orders of the nociceptive stimuli x 8 SOA’s. Trials were presented randomly within each block of stimulation. The visual cues were not spatially informative and the location of any forthcoming nociceptive stimulus could thus not be predicted by the cue.

Participants were instructed to keep their gaze on the fixation LED and to indicate which hand was stimulated first in two blocks, and which hand was stimulated second in the other two blocks. By using both a “*Which came first*?” and a “*Which came second*?” task, we were able to control for response bias (that is, the tendency of participants to respond with the side on which the unilateral cue had been presented; see [28, 39–41]). The instruction was alternated between blocks of trials and the order of presentation was counterbalanced across participants. Participants’ responses were provided verbally and registered by the experimenter by pressing one of two keys on a keyboard. Participants were explicitly instructed to tell which *hand* was stimulated first/second, irrespective of the side of space in which their hand was stimulated. After the participants had made their response, the fixation LED was turned off. The verbal responses were encoded by the experimenter. After 1000 ms, the next trial started. The experiment took on average 60 minutes.

#### 2.2.4. Measures

**Fig 3:**
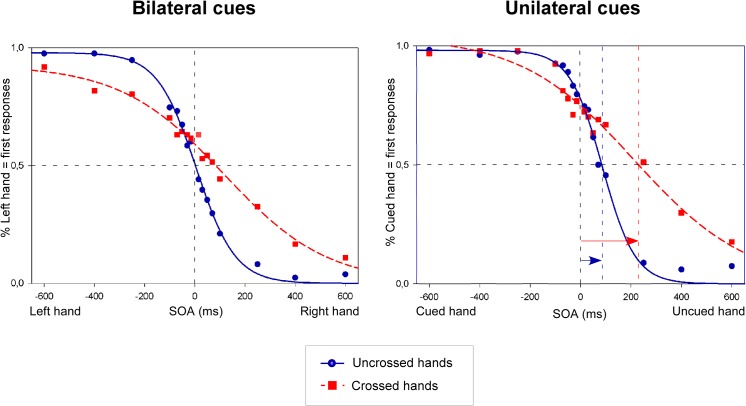
Nociceptive temporal order judgments (TOJs) in Experiment 2. The figures illustrate the fitted curves from cumulative data from 17 participants. Trials were either associated with bilateral cues (left side of the figure), or with an unilateral cue (right side of the figure), and with an uncrossed (blue solid line) or a crossed (red dashed line) posture. Data are plotted as the mean proportion of *left hand first responses* (on the Y-axis; left side of figure, bilateral cues) or *cued hand first responses* (on the Y-axis; right side of figure, unilateral cues), as a function of the stimulus onset asynchronies (SOA) (on the X-axis). On the X-axis, for the bilateral cue conditions, the negative values of the SOAs indicate that the left hand was stimulated first, and the positive values indicate that the right hand was stimulated first. In the unilateral cue condition, negative values indicate that the cued hand was stimulated first, while positive values indicate that the uncued hand was stimulated first. The vertical dashed lines coincide with the PSS values. As in Experiment 1, the curves in the unilateral cue conditions were shifted toward the uncued side both for the uncrossed and the crossed posture. This indicates that the nociceptive stimulus on the uncued hand had to be presented several milliseconds before the stimulus on the cued hand to have an equal chance to be perceived first. This effect was even stronger for the crossed posture.

For each participant, and for each SOA of the 4 within-participant conditions (bilateral vs. unilateral cues x uncrossed vs. crossed posture), the proportion of trials on which participants perceived the cued hand as being stimulated first was calculated (see Fig 3). In order to increase the number of trials per condition, we merged the data over the variable *Task* (Which first? vs. Which second?), as this variable was not of primary interest, and previous studies with a similar paradigm had shown that the task participants have to perform, has no significant influence on the TOJ performance [5]. PSS and JND values were calculated from these proportions identically to the first experiment. In order to control whether the side at which the visual cue was presented could have influenced the PSS values in unilateral cue trials, we did a separate analyses including *Side of the visual stimulation* as a factor. These analyses showed that merging PSS values for cues presented on the left and the right side of space will not distort the results (see data in S1 Appendix).

#### 2.2.5. Analyses

In this experiment PSS values that exceeded the maximum SOA (± 600, instead of twice the maximum SOA) were excluded from the data, together with their corresponding JND values, and were considered as missing values. This was done, because taking twice the maximum SOA as cut-off would mean that participants could have PSS values as large as 1200, which we considered to be too extreme. The difference in missing values between the uncrossed and the crossed posture condition was compared using a chi-squared test for equality of proportions. We evaluated whether the PSS values were significantly different from 0 using one-sample t-tests. In order to compare the PSS across the different experimental conditions, results were analyzed using the linear mixed effects models as implemented in the R package “Linear and Nonlinear Mixed Effects Models” [31]. The first outcome variable was the Point of Subjective Simultaneity (PSS). The independent variables were the *Laterality* (unilateral/bilateral), and the *Posture* (uncrossed/crossed). The same analyses approach as for the first experiment was used. The models are shown in the supporting information (Tables D-F in S1 File).

The same method was used to assess the influence of the different experimental conditions on the JND. The models are shown in the supporting information (Tables J-L in S1 File).

## Results

### 3.1. Intensity of the nociceptive stimuli

The mean current intensities used during Experiment 1 were 0.58 ± 0.20 mA and 0.58 ± 0.21 mA for the left and right hands respectively. During Experiment 2, the current intensities were 0.58 ± 0.23 mA and 0.55 ± 0.23 mA for the left and right hand respectively. The differences between the left and the right hand were not significant (Experiment 1: t(20) = 0.08, *p* = 0.94; Experiment 2: t(16) = 1.0, *p* = 0.33). The mean self-reported intensities (VAS) were, during Experiment 1, 3.13 ± 1.68 for the left hand and 3.36 ± 1.53 for the right hand (t(20) = -2.37, *p* = 0.03). During Experiment 2 the self-reported intensities were 4.07 ± 1.66 for the left hand, and 3.82 ± 1.40 for the right hand (t(16) = 1.92, *p* = 0.07). The analyses revealed that the self-reported intensities were significantly different for the left and the right hand in both experiments, but such a difference was marginal (0.23 for Experiment 1, and 0.25 for Experiment 2), and did not affect the results.

### 3.2. Missing values

In Experiment 1, 28 out of 168 (17%) of the values were excluded; 25 of these were from the crossed posture condition. A chi-squared test indicated that the proportion missing values was significantly larger for the crossed posture (30%) than for the uncrossed posture (4%) (χ^2^(1,168) = 18.9; *p* < 0.001). In Experiment 2, 6 out of 68 (9%) of the values were excluded; all of these were from the crossed posture condition. A chi-squared test indicated that the proportion missing values was significantly larger for the crossed posture (18%) than for the uncrossed posture (0%) (χ^2^(1,68) = 6.58; *p* = 0.03). These results show a larger number of missing values for the crossed hands condition in both experiments. In order to account for the large amount of missing values in the crossed posture condition, two further analyses were conducted to check whether results remain the same when the participants who performed poorest were removed from the analyses. Removing these participants did not substantially change results (see data in S2 Appendix).

### 3.3. PSS

**Fig 4:**
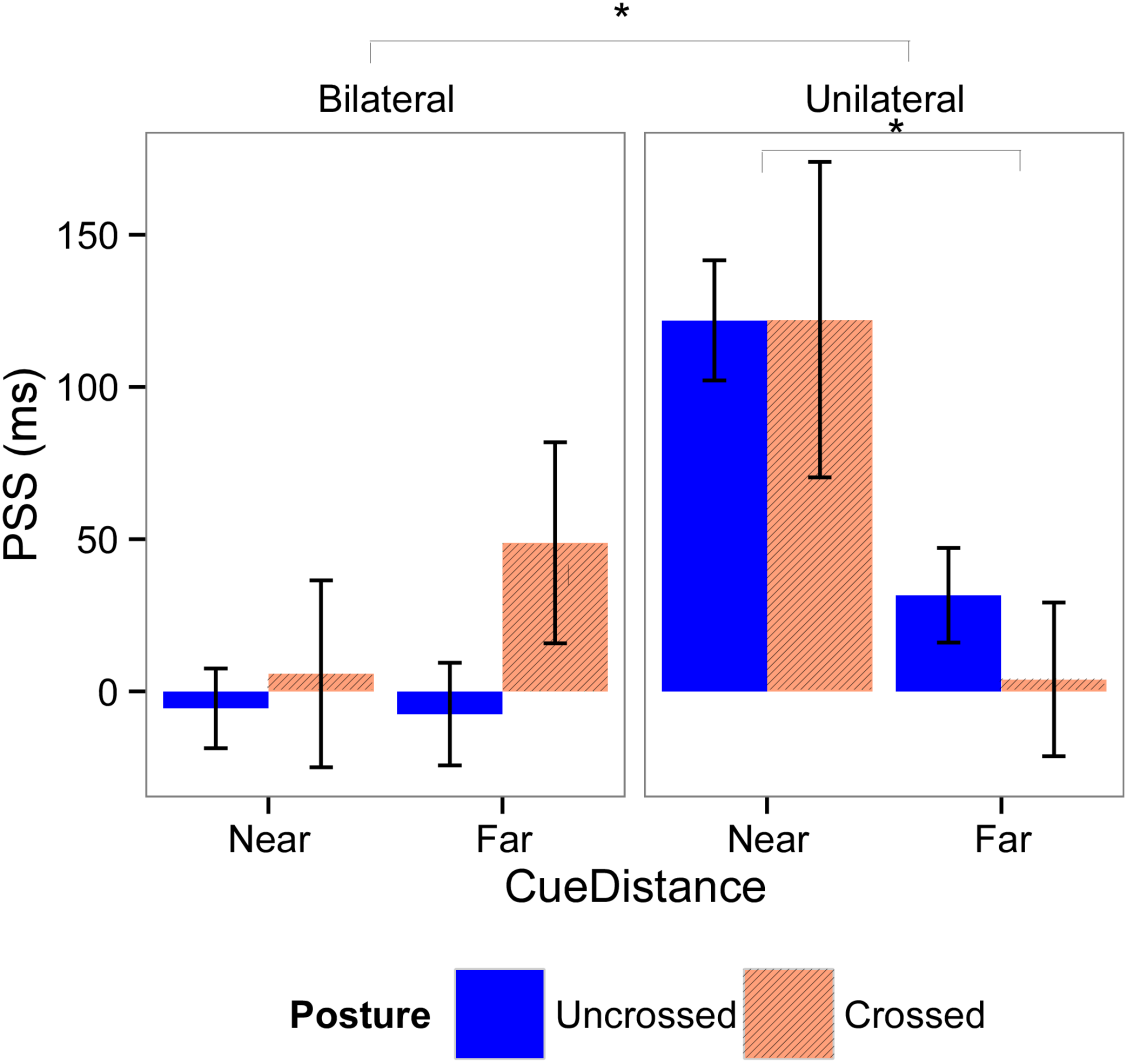
Means and standard errors for the point of subjective simultaneity (PSS) for Experiment 1. PSS values were calculated according the laterality of the visual cues (left graphic, bilateral cues; right graphic, unilateral cue), distance of the cues (left part of the graphics, near; right part of the graphics, far), and posture of the hands (blue bars, uncrossed; red bars, crossed). Significant effects are indicated with an asterisk.

For Experiment 1, mean PSS values are displayed in Fig 4. In the unilateral cue conditions, the one-sample t-test revealed that for the uncrossed posture, all PSS values were significantly different from 0 (all t > 2.0, all *p* < 0.05), suggesting a significant bias in the temporal order judgment. For the crossed posture, the PSS values were significantly different from 0 when cues were presented near the participants (t(9) = 2.36, *p* = 0.04), but not when cues were presented far from the participants (t(14) = 0.16, *p* = 0.88). In the bilateral cue condition, none of the PSS values were significantly different from 0, neither for the uncrossed posture (all t < 0.45, all *p* > 0.65), nor for the crossed posture (all t <1.5, all *p* > 0.15). This result indicates that the PSS is only biased by the presence of an unilateral visual cue, and never in the presence of bilateral cues.

The linear mixed effects model that demonstrated the best fit with our data includes all fixed factors, the interaction effect between *Laterality* and *Cue Distance*, and a random subject-based intercept. In this final model, we found a main effect of *Laterality* (F(1,122.76) = 24.06; *p* < 0.001; *β* = 0.57), indicating that the PSS was more positive when cues were presented unilaterally than when they were presented bilaterally. Moreover, there was a significant interaction effect between *Laterality* and *Cue Distance* (F(1,119.24) = 12.38; *p* < 0.001, *β* = -1.24). Post-hoc analyses show that there was no significant effect of *Cue Distance* in bilateral trials (χ^2^(1, N = 21) = 0.63, *p* = 0.43), however *Cue Distance* had a significant effect in unilateral trials (χ^2^(1, N = 21) = 16.36, *p* < 0.001): in these trials the PSS was more positive when cues were presented near, than when they were presented far. The main effect of *Posture* was not significant (F(1,123.70) = 0.47, *p* = 0.49, *β* = 0.05), showing that the cued hand was prioritized, no matter whether the hands were uncrossed or crossed. The main effect of *Cue Distance* was not significant (F(1,117.5) = 0.62, *p* = 0.43, *β* = 0.08).

**Fig 5:**
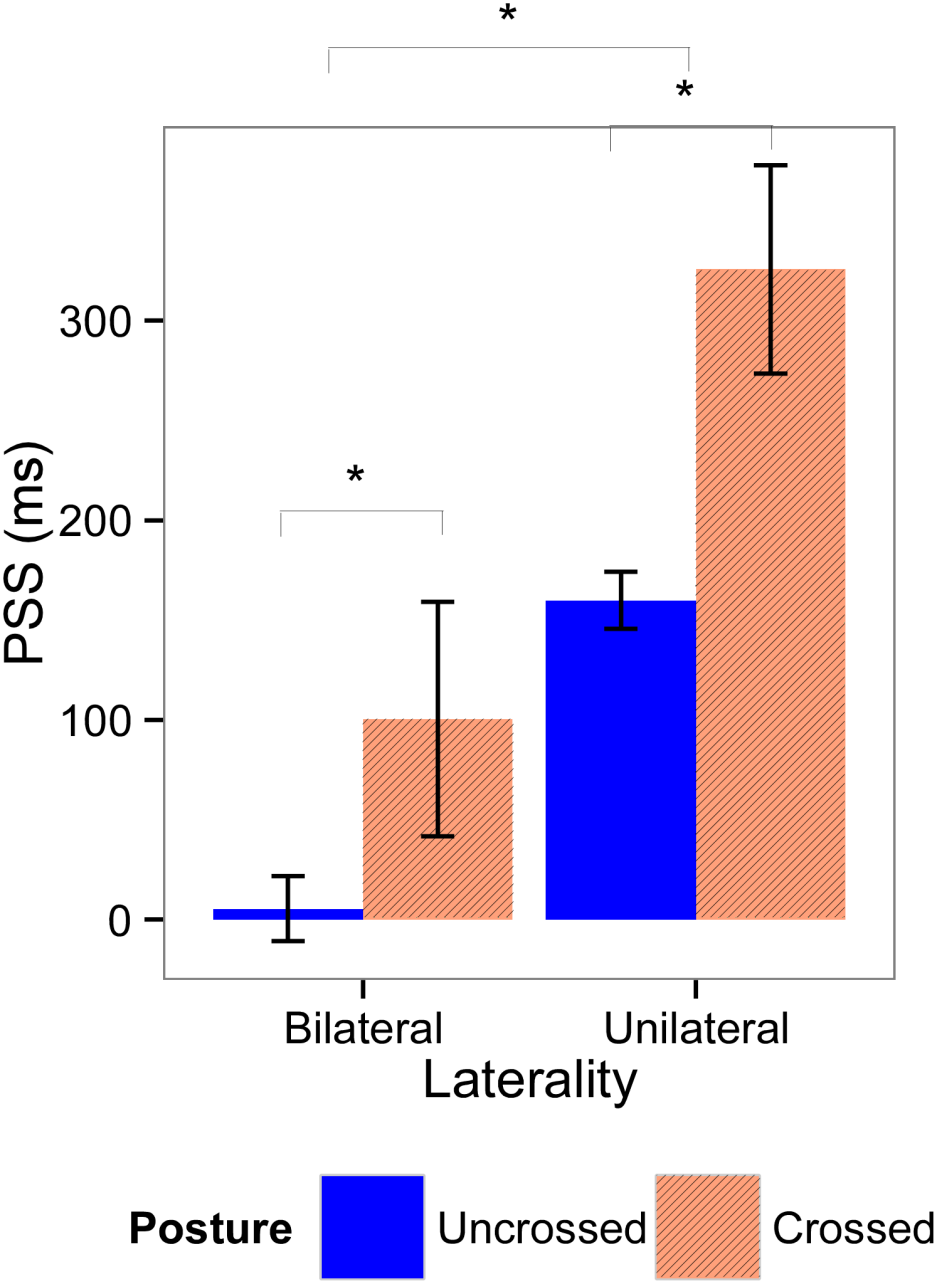
Means and standard errors for the PSS for Experiment 2. PSS values were calculated according the laterality of the visual cues (left graphic, bilateral cues; right graphic, unilateral cue) and posture of the hands (blue bars, uncrossed; red bars, crossed). Significant effects are indicated with an asterisk.

For Experiment 2, mean PSS values are displayed in Fig 5. The t-tests showed that the PSS values were significantly different from 0 when cues were presented unilaterally (all t > 6.0, all *p* < 0.001), whereas no bias was induced when cues were presented bilaterally (all t < 2.0, all *p* > 0.10).

The model that demonstrated the best fit with our data includes all fixed factors and a random subject-based intercept. In this model, there was a main effect of *Laterality* (F(1,45.48) = 22.09, *p* < 0.001, *β* = 0.51), indicating that PSS values were larger when cues were presented unilaterally, than when they were presented bilaterally. Moreover, there was a main effect of *Posture* (F(1, 45.48) = 10.21, *p* = 0.002, *β* = 0.34), indicating that PSS values were larger when hands were crossed than when hands were uncrossed. However, in both cases the PSS is positive, indicating an attentional bias towards the cued hand irrespective of posture.

### 3.4. JND

**Fig 6:**
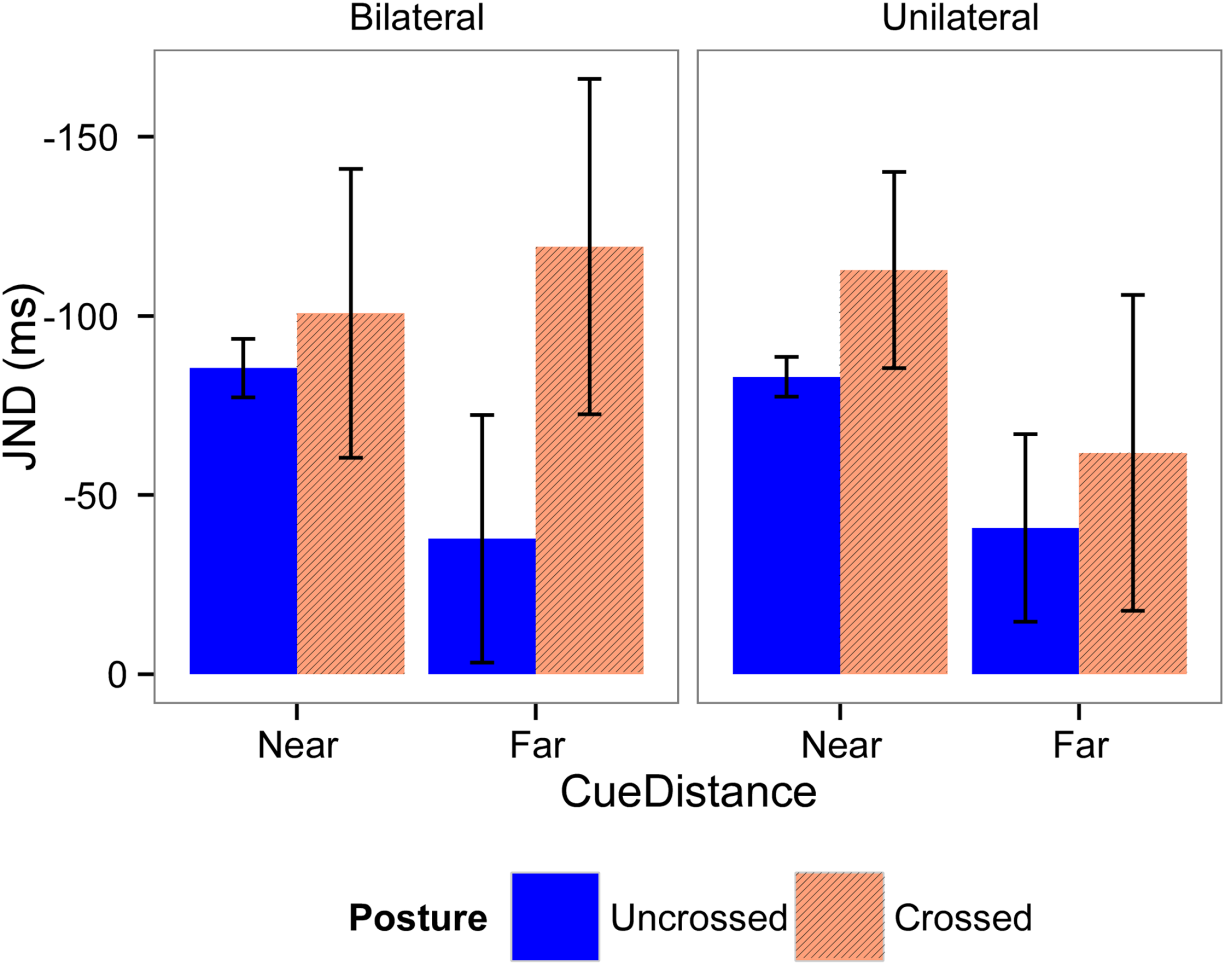
Means and standard errors for the JND for Experiment 1. JND values were calculated according the laterality of the visual cues (left graphic, bilateral cues; right graphic, unilateral cue), distance of the cues (left part of the graphics, near; right part of the graphics, far), and posture of the hands (blue bars, uncrossed; red bars, crossed). There were no significant differences between conditions.

**Fig 7:**
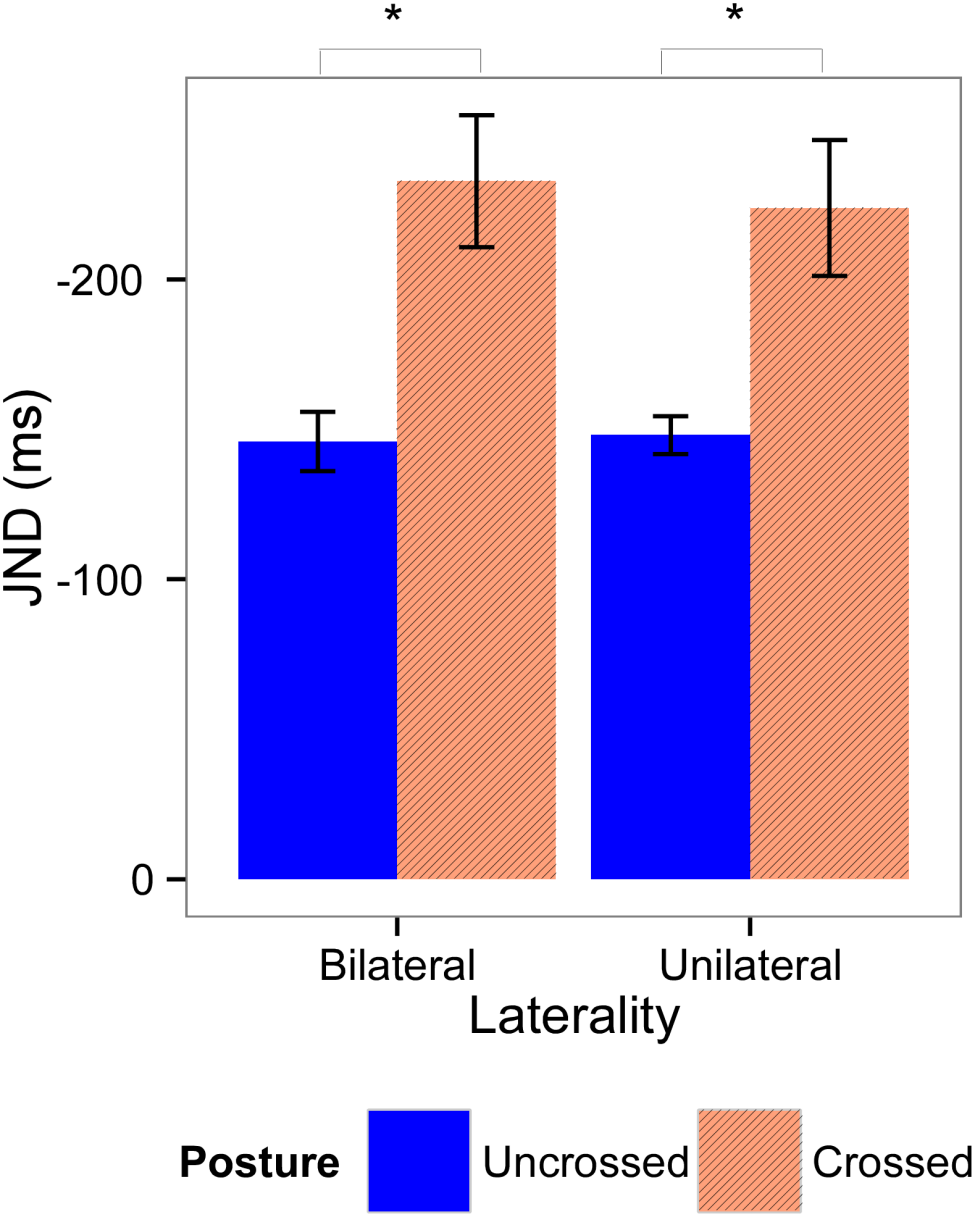
Means and standard errors for the JND for Experiment 2. JND values were calculated according the laterality of the visual cues (left graphic, bilateral cues; right graphic, unilateral cue) and posture of the hands (blue bars, uncrossed; red bars, crossed). Significant effects are indicated with an asterisk.

Mean JND values for Experiments 1 and 2 are shown in Figs 6 and 7 respectively. For Experiment 1, the model with the best fit included all fixed factors, a random subject-based intercept, and a random effect for *Cue Distance* and *Posture*. In this model, there were no significant effects present (Table I in S1 File). For Experiment 2, the model chosen included all fixed factors, a random subject-based intercept, and a random effect for *Posture*. This model demonstrated a significant main effect of *Posture* (F(1,16.09) = 18.33, *p* < 0.001, *β* = -0.64), showing that participants’ temporal order judgments were less accurate when their hands were crossed, than when their hands were uncrossed.

## Discussion

This study investigated whether nociceptive stimuli are mapped according to a spatiotopic frame of reference, and more particularly a peripersonal frame of reference that takes into account both the influence of external sensory events near the body, and the relative position of the stimulated body part in external space. Two TOJ studies were conducted in which pairs of nociceptive stimuli were presented, one stimulus applied to either hand at various SOAs. The nociceptive stimuli were shortly preceded by visual cues, and the influence of these cues on the TOJ performance was assessed. The crucial manipulation in the present experiments was that participants’ posture was changed across the experimental blocks. In some blocks participants’ hands were uncrossed, whereas in other blocks participants were asked to cross their hands across the sagittal midline of the body. The results of both experiments demonstrated that the temporal order of nociceptive stimuli was not merely influenced by the position of the nociceptive stimuli on the body, but mostly by the position of the stimulated hand in external space. Indeed, PSS values were shifted towards the uncued side of space, and these shifts were influenced by the relative posture. In other words, a left visual cue prioritized the perception of nociceptive stimuli applied to the left hand in the uncrossed posture, but to the right hand in the crossed posture, and vice versa. In Experiment 1, we further showed that the influence of the cues was smaller when they were presented far in front of the participants’ hand as compared to when they were presented at its close proximity [5]. In addition, the temporal order judgments were less accurate in the crossed than in the uncrossed posture condition, as witnessed by the larger amount of errors and the larger JND in the former than in the latter condition.

The localization of nociceptive stimuli is an important function of the nociceptive system. It not only enables us to detect which part of the body is damaged, but also to detect the source of the damage in the external space. Therefore, a finely-tuned localization of noxious stimuli will help to react adequately against potentially threatening objects. In daily life, we are constantly moving, so that our limbs can be positioned in different locations, also at the opposite side of space. Therefore, a somatotopic frame of reference is insufficient to localize nociceptive stimuli, and the body space has to be remapped into a spatiotopic frame of reference, which takes into account the relative position of the body limbs in external space. Several studies have found evidence for the existence of a spatiotopic reference frame for the localization of both tactile [9–12] and nociceptive stimuli [21, 22] using the crossing hands procedure. In the two present studies we wanted to go one step further by showing that nociceptive stimuli are mapped in a peripersonal frame of reference that also integrates the occurrence of external objects presented near the body. For tactile stimuli, several studies using a crossmodal congruency task performed with uncrossed and crossed posture, already showed that visual cues prioritize the tactile stimulation applied to the hand lying in the cued side of space [13–17]. This indicates that representations of visuotactile peripersonal space are updated when hands are crossed over the body midline. In the present studies we extended these findings to nociceptive stimuli. We showed that the influence of visual stimuli on nociceptive processing is space-based, i.e. the visual cues prioritized the processing of the nociceptive stimuli applied to the hand located in the cued side of space, irrespective of its posture. Moreover, in Experiment 1, we found that the influence of the visual stimuli is larger when they were presented near the hands of the participants as opposed to when they were presented far away. This is in accordance with previous findings showing that the processing of nociceptive stimuli is affected by visual cues presented in peripersonal space, but to a lesser extent by cues presented in extrapersonal space [5]. Taken together, these results provide strong evidence for the existence of a peripersonal frame of reference for the localization of nociceptive stimuli. A peripersonal frame of reference allows for the construction of a stable perception of external space, which is necessary to react quickly and efficiently to stimuli in the environment. Peripersonal space can be seen as a kind of safety margin around the body that is scanned for potentially threatening stimuli and that enables us to detect, localize and react against these stimuli [42, 43].

It is interesting to note that, in Experiment 2, the PSS values were larger when the hands were crossed as compared to conditions during which the hands were uncrossed. This could suggest that the dissociation generated between somatotopic and spatiotopic frames of reference by the crossed posture facilitated the influence of visual stimuli on the spatial processing of nociceptive stimuli. However, such a hypothesis was not supported by the data from Experiment 1, and therefore needs further demonstration.

The JND gives an indication of the temporal sensitivity of participants’ judgments. In previous studies a crossed hands deficit is consistently found both in studies using tactile [9–12] and nociceptive stimuli [21]. These studies show larger JND values, and thus decreased temporal sensitivity when hands are crossed over the body midline. It is argued that the decreased performance resulting from crossing the hands can be explained by a competition between a somatotopic and a spatiotopic frame of reference [9, 21]. The right hand most commonly occupies the right side of space, and the left hand occupies the left side of space. When the posture is changed, a process of remapping is thus required to correct the position. This remapping process takes time, which explains why the ability to discriminate the order in which the hands are stimulated is impaired at shorter intervals: the position of the first stimulus is still being processed, while the second stimulus is presented [9, 21]. Based on reversal errors at smaller intervals, Yamamoto et al. [9] suggested that this remapping process takes around 300 ms to complete. However inter-subject variability in the time this remapping process takes might be present.

In the present study, we only found a significant difference in the JND between the different postures in Experiment 2, but not in Experiment 1. A possible explanation for this apparent discrepancy is the fact that, in Experiment 1, we had to exclude significantly more values in the crossed (30%) than in the uncrossed condition (3%). Doing this might have artificially reduced the difference in JND between the uncrossed and the crossed posture. However, keeping these values in the analyses made no sense, as the PSS values in these conditions were extreme (e.g. 1.19 x 10^18^), indicating that participants were unable to perform the task. Indeed, the larger amount of excluded trials in the crossed hands condition indicates that the posture of the hands affected the ability of the participants to judge the temporal order of the nociceptive stimuli. For these participants the remapping process might have been incomplete even at the largest SOAs (+/- 200 ms), making it impossible for them to complete the task successfully when hands were crossed. This result is in line with the suggestion of Yamamoto et al. [9] that the remapping process takes around 300 ms. Moreover it is in line with a study of Azanon and Soto-Faraco [43], in which the time-course of the remapping process from a somatotopic to a spatiotopic frame of reference was investigated using a crossmodal cuing paradigm. Participants held their arms crossed over the body midline, and were instructed to judge the vertical position (up vs. down) of light flashes. These flashes were preceded by irrelevant tactile cues with varying cue target onset asynchronies (CTOA). They found that at short CTOAs the spatial cuing effect corresponded to somatotopic representations, demonstrated by the fact that touches to the left hand (placed in the right hemisphere) facilitated processing of left hemispace visual events and vice versa. This pattern reversed after 200 ms so that tactile cues facilitated the processing of targets presented at the same external location. In a subsequent study they showed that these crossmodal links are spatially specific, as they appear to be stronger in peripersonal space than in extrapersonal space. This study reveals the time-course of the encoding of events in tactile space, from a somatotopic frame of reference, reflecting the neural organization in the primary somatosensory cortex (SI), to an external representation of space, enabling orienting behaviors. This remapping process would not start before 60 ms after stimulus application, and would be completed after 180 to 360 ms.

In Experiment 2 larger SOAs were used (up to 600 ms) to make the task easier. As expected, we had to exclude less values in the crossed hands condition (18%), indicating that for most participants, the remapping process had completed at the largest SOAs. Of interest, we now found that the JND was significantly higher when hands were crossed than when hands were uncrossed, indicating reduced temporal sensitivity when hands were crossed. Moreover, when we excluded the subjects who performed poorest in Experiment 1, a marginally significant effect of posture was also found, again demonstrating a reduced temporal sensitivity in the crossed posture condition. Therefore, we can conclude that our pattern of results is in line with the previous studies [9–12, 21], showing that changing the posture affects the ability to process the spatial location of somatosensory stimuli, including nociceptive stimuli. It confirms our prediction according to which the spatial perception of nociceptive stimuli is made according to a spatiotopic mapping system.

The present study also points out the importance of spatial perception for the understanding of the pathophysiology and the treatment of chronic pain. Some chronic pain patients, more particularly patients with CRPS, show impairment in body representation and spatial perception (for a review, see [44]). These patients tend to ignore or have an altered mental representation of the affected limb, and movements are smaller and less frequent [45–48]. Using a TOJ task, Moseley et al. [49] found that CRPS patients prioritize the perception of tactile stimuli applied to the unaffected arm to the detriment of those presented to the affected arm. Interestingly, when participants were asked to cross their arms, results were reversed: the perception of tactile stimuli on the affected arm was prioritized over the perception of those on the unaffected arm. In addition, crossing the hands also affected their accuracy in reporting the temporal order of the tactile stimuli. These data suggest that the impairment in these patients is not linked to the affected limb itself, but rather to the side of space in which the limb normally resides. The presence of chronic pain and other CRPS-related symptoms can thus alter the ability to perceive the body, not only according to somatotopic but also according to spatiotopic frames of reference [44]. Furthermore, some studies showed that manipulating the spatial perception of these patients can alleviate pain [50, 51]. These studies used prism adaptation to shift the visual field of CRPS patients towards the unaffected side, resulting in an after-effect towards the affected limb. They found that this relieved pain and autonomic dysfunction, and that it reduced their pathological perceptions of the body midline. These studies illustrate that some somatosensory deficits are not explained by somatotopic frames of reference but rather by space-based frames of reference. Moreover, they suggest that manipulating the spatial perception could be a potential rehabilitation technique for some chronic pain patients.

It has to be noted that based on the present results, we cannot be sure whether the crossmodal shifts in the PSS between vision and nociception result from exogenous shifts of spatial attention from one space (i.e. external proximal space) to another space (i.e. bodily space), or from intrinsic multisensory integration [4]. In the former case, salient but spatially non-predictive visual cues could have attracted multisensory spatial attention to its location, leading to a faster processing of the forthcoming nociceptive target. Multisensory integration on the other hand occurs when two different-modality stimuli that are presented around the same time and place are integrated to form a unified perceptual object, instead of a collection of unrelated sensations. This would result from an additive sensory response from specialized neurons that respond to stimuli of both modalities [52]. Another mechanism relies on the existence of multimodal neurons with multiple receptive fields to code the location of sensory inputs from different modalities. The non-somatic (i.e., visual and auditory) receptive fields extend the region of the somatic (i.e., tactile) receptive field into the immediate adjacent space. Therefore, these neurons respond both to the stimuli applied to a specific area of the skin surface and to stimuli appearing in the space proximal to the stimulated body area [20, 53]. Further studies are needed to dissociate these different mechanisms in the spatial perception of nociceptive stimuli.

One could argue that the judgment bias induced by proximal visual stimuli on the processing of nociceptive stimuli does not fully support the hypothesis that nociceptive inputs can be remapped according to a spatiotopic frame of reference. Indeed, because the spatial position of visual stimuli is primarily coded by the cortical projections of the retinas, one should also evidence how visual inputs are remapped from retinotopic to spatiotopic frames of reference. More specifically, further studies are needed to understand how, during crossmodal interaction between somatosensory and visual inputs, visual stimuli are remapped according to their proximity to body parts into a body-centered representation of external space. However, this does not preclude that previous and present data support the hypothesis according to which nociceptive mapping can be spatiotopic. First, our previous studies [5] showed that changing gaze fixation, and thus changing the position of the visual stimulus on the retina, does not change the results. Second, judgments’ sensitivity, as indexed by JND, was affected by the posture of the hands, both with (present data) or without [21] visual cues, suggesting that nociceptive mapping depends on the relative position of the body limb in external space (see also [6–12]).

Finally, despite the procedure applied to match intensities of the nociceptive stimuli applied to left and right hands, the strict equivalence between the subjective perception of the intensities between the two hands could not always be achieved. Such differences were very marginal (0.23 to 0.25 cm on a rating scale of 10 cm) and could not have affected the results. Indeed, the results show that the PSS, and, therefore, the judgment biases, were not affected by the hand on which the nociceptive stimuli were perceived as the most intense (for instance, in the bilateral conditions, the PSS are never significantly different from 0), but only by the side of the visual cues.

## Supporting Information

S1 File Linear mixed effects models. Table A-L.
(PDF)

S1 Appendix Analyses to control for effects of the side of the visual stimulation. (PDF)

S2 Appendix Sensitivity analyses. (PDF)
