# Supplementary material for: From a Somatotopic to a Spatiotopic Frame of Reference for the Localization of Nociceptive Stimuli
Source: PLoS One. 2015 Aug 28;10(8):e0137120. doi: 10.1371/journal.pone.0137120 (PMC4552762; doi:10.1371/journal.pone.0137120)
Supplement: S1 File — Table A-L. (PDF) [file pone.0137120.s001.pdf]

S1 File. Linear Mixed Effect Models.

For the behavioral measures (PSS and JND), we started with a full model of the fixed effects. We then added the random effects that were necessary, based on Akaike's information criterion (AIC, Sakamoto, Ishiguro, & Kitagawa, 1986), and the likelihood-ratio test. Subsequently, we determined if interactions between the fixed effects should be included. As we were interested in all included variables, fixed effects were never removed from the model. At each step the most parsimonious model was selected, that, at the same time, performed best at predicting the dependent variables. When the fixed effects were determined, the final model was refitted with REML estimation and the relevant contrasts were calculated. For each behavioral measure, the three fitting steps are presented below. For each step, the AIC, the χ^2 for the relevant model comparisons, and the corresponding p-values are presented. The final table for each measure shows the Anova table, and the parameter estimates with their corresponding t-values.

**Model**	Test	Random	AIC	Df	χ^2	*p*-value
**1**	Initial fit	1	1700.2	10		
**2**	Random Laterality (1 vs. 2)	1 + Laterality	1701.9	12	$\chi^2(2) = 2.31$	0.31
**3**	Random Cue Distance (1 vs. 3)	1 + Cue Distance	1704	12	$\chi^2(2) = 0.0036$	0.998
**4**	Random Posture (1 vs. 4)	1 + Posture	1700.9	12	$\chi^2(2) = 3.30$	0.19

S1 Table A. Step 1 Experiment 1 – PSS. Determine random effects structure, all models have 'subject' as random intercept. Decision: no random effects added, keep model 1.

Model	Test	Fixed	AIC	Df	χ^2	*p*-value
**1**	Initial fit	Laterality*Posture*Cue Distance	1700.2	10		
**2**	Remove three-way interaction (1 vs. 2)	Laterality*Cue Distance + Laterality*Posture + Posture*Cue Distance	1699.4	9	$\chi^2(1) = 1.16$	0.28
**3**	Remove interaction with Posture (2 vs. 3)	Laterality*Cue Distance + Posture	1697.7	7	$\chi^2(2) = 2.30$	0.32
**4**	Remove interaction with Laterality (2 vs. 4)	Cue Distance*Posture + Laterality	1709.7	7	$\chi^2(2) = 14.35$	<0.001
**5**	Remove interaction with Cue Distance (2 vs. 5)	Laterality*Posture + Cue Distance	1707.7	7	$\chi^2(2) = 12.29$	0.002
**6**	Remove all interactions (3 vs. 6)	Laterality + Posture + Cue Distance	1708.0	6	$\chi^2(1) = 12.27$	<0.001

S1 Table B. Step 2 Experiment 1 – PSS. Determine fixed effects – Trim down the model. Decision: choose model 3 with the interaction between Laterality * Cue Distance

**Effects**	F	Df1	Df2	*p*	*β*
**Laterality**	24.06	1	122.76	<0.001	0.57
**Cue Distance**	0.62	1	117.50	0.43	0.08
**Posture**	0.47	1	123.70	0.49	0.05
**Laterality*Cue Distance**	12.38	1	119.24	<0.001	-1.24

	B	**SE(B)**	**t**
**Intercept**	-5.612	18.13	-0.31
**Laterality**	123.143	25.027	4.92
**Cue Distance**	18.19	22.99	0.79
**Posture**	11.82	17.21	0.69
**Laterality*Cue Distance**	-120.43	34.17	-3.52

S1 Table C. Step 3 Experiment 1 – PSS. Test final model.

Model	Test	Random	AIC	Df	χ^2	*p*-value
**1**	Initial fit	1	808.01	6		
**2**	Random Laterality (1 vs. 2)	1 + Laterality	810.70	8	$\chi^2(2) = 1.32$	0.52
**3**	Random Posture (1 vs. 3)	1 + Posture	809.73	8	$\chi^2(2) = 2.28$	0.32

S1 Table D. Step 1 Experiment 2 – PSS. Determine random effects structure, all models have 'subject' as random intercept. Decision: choose model 1 without additional random effects.

**Model**	Test	Fixed	AIC	Df	χ^2	*p*-value
**1**	Initial fit	Laterality*Posture	808.01	6		
**2**	Remove interaction (1 vs. 2)	Laterality + Posture	806.86	5	$\chi^2(1) = 0.85$	0.36

S1 Table E. Step 2 Experiment 2 – PSS. Determine fixed effects – Trim down the model. Decision: Choose model 2 without interaction.

**Effects**	F	Df1	Df2	*p*	*β*
**Laterality**	22.09	1	45.48	<0.001	0.51
**Posture**	10.21	1	45.48	0.002	0.34

	B	**SE(B)**	**t**
**Intercept**	-10.14	32.83	-0.309
**Laterality**	185.60	39.34	4.718
**Posture**	126.17	39.34	3.207

S1 Table F. Step 3 Experiment 2 – PSS. Test final model.

**Model**	Test	Random	AIC	Df	χ^2	*p*-value
**1**	Initial fit	1	1786.4	10		
**2**	Random Laterality (1 vs. 2)	1 + Laterality	1790.4	12	$\chi^2(2) = 0$	1
**3**	Random Cue Distance (1 vs. 3)	1 + Cue Distance	1776.7	12	$\chi^2(2) = 13.72$	0.001
**4**	Random Cue Distance and Posture (3 vs. 4)	1 + Cue Distance + Posture	1767.3	15	$\chi^2(3) = 15.39$	0.002
**5**	Random Cue Distance, Posture and Laterality (4 vs. 5)	1 + Cue Distance + Posture + Laterality	1774.6	19	$\chi^2(4) = 0.69$	0.95

S1 Table G. Step 1 Experiment 1 – JND. Determine random effects structure, all models have ‘subject’ as random intercept. Decision: choose model 6 with Cue Distance and Posture as random effects.

Model	Test	Fixed	AIC	Df	χ^2	*p*-value
**1**	Initial fit	Laterality*Posture*Cue Distance	1767.3	15		
**2**	Remove three-way interaction (1 vs. 2)	Laterality*Cue Distance + Laterality*Posture + Posture*Cue Distance	1766.6	14	$\chi^2(1) = 1.31$	0.25
**3**	Remove interaction with Posture (2 vs. 3)	Laterality*Cue Distance + Posture	1765.4	12	$\chi^2(2) = 2.78$	0.25
**4**	Remove interaction with Laterality (2 vs. 4)	Cue Distance*Posture + Laterality	1765.0	12	$\chi^2(2) = 2.31$	0.32
**5**	Remove interaction with Cue Distance (2 vs. 5)	Laterality*Posture + Cue Distance	1765.1	12	$\chi^2(2) = 2.41$	0.30
**6**	Remove all interactions (2 vs. 6)	Laterality + Posture + Cue Distance	1764.4	11	$\chi^2(3) = 3.72$	0.29

S1 Table H. Step 2 Experiment 1 – JND. Determine fixed effects – Trim down the model. Decision: choose model 6 without interactions.

**Effects**	F	Df1	Df2	*p*	*β*
**Laterality**	0.73	1	85.10	0.40	0.11
**Cue Distance**	0.73	1	19.88	0.40	0.06
**Posture**	1.23	1	19.17	0.28	-0.13

	B	**SE(B)**	**t**
**Intercept**	-83.75	17.31	-4.84
**Laterality**	15.32	17.83	0.859
**Cue Distance**	28.67	33.51	0.856
**Posture**	-34.82	31.16	-1.12

S1 Table I. Step 3 Experiment 1 – JND. Test final model

Model	Test	Random	AIC	Df	χ^2	*p*-value
**1**	Initial fit	1	692.00	6		
**2**	Random Laterality (1 vs. 2)	1 + Laterality	695.46	8	$\chi^2(2) = 0.54$	0.76
**3**	Random Posture (1 vs. 3)	1 + Posture	666.37	8	$\chi^2(2) = 29.63$	<0.001

S1 Table J. Step 1 Experiment 2 – JND. Determine random effects structure, all models have 'subject' as random intercept. Decision: choose model 3 with Posture as random effect.

**Model**	Test	Fixed	AIC	Df	χ^2	*p*-value
**1**	Initial fit	Laterality*Posture	666.37	8		
**2**	Remove interaction (1 vs. 2)	Laterality + Posture	665.35	7	$\chi^2(1) = 0.98$	0.32

S1 Table K. Step 2 Experiment 2 – JND. Determine fixed effects – Trim down the model. Decision: choose model 2 without interaction.

**Effects**	F	Df1	Df2	*p*	*β*
**Laterality**	1.07	1	27.44	0.31	-0.06
**Posture**	18.33	1	16.09	<0.001	-0.64

	B	**SE(B)**	**t**
**Intercept**	-142.90	8.69	-16.44
**Laterality**	-8.19	7.90	-1.037
**Posture**	-92.89	21.69	-4.28

S1 Table L. Step 3 Experiment 2 – JND. Test final model.
