# Supplementary material for: From a Somatotopic to a Spatiotopic Frame of Reference for the Localization of Nociceptive Stimuli
Source: PLoS One. 2015 Aug 28;10(8):e0137120. doi: 10.1371/journal.pone.0137120 (PMC4552762; doi:10.1371/journal.pone.0137120)
Supplement: S1 Appendix — (PDF) [file pone.0137120.s002.pdf]

S1 Appendix. Analyses to control for effects of the side of the visual stimulation.

To check whether an effect of the side at which the visual cue was presented existed, a separate analysis was performed on the unilateral cue trials. In Experiment 1, we looked at the PSS as a function of the *Side of the visual cue,* the *Posture,* and the *Cue Distance*. There was a main effect of *Side* ($F(1,110.43) = 5.56$, $p = 0.02$), indicating that PSS values were overall higher when the left side was cued, than when the right side was cued. There was also a main effect of *Cue Distance* ($F(1,113.30) = 29.92$, $p < 0.001$), indicating that the PSS values were higher when cues were presented near the participants than when they were presented far away. The main effect of *Posture* was not significant ($F(1,116.49) = 0$, $p = 0.99$). However, as there was no interaction effect involving the *Side of the visual cue,* merging data for left and right cues will not distort results.

In Experiment 2, we looked at the PSS as a function of the *Side of the visual cue*, and the *Posture*. There was a main effect of *Posture* ($F(1,42.62) = 10.77$, $p = 0.002$), indicating that PSS values were higher when hands were crossed than when they were uncrossed. The main effect of *Side* was not significant ($F(1,36.80) = 0.33$, $p = 0.57$).
