# Supplementary material for: From a Somatotopic to a Spatiotopic Frame of Reference for the Localization of Nociceptive Stimuli
Source: PLoS One. 2015 Aug 28;10(8):e0137120. doi: 10.1371/journal.pone.0137120 (PMC4552762; doi:10.1371/journal.pone.0137120)
Supplement: S2 Appendix — (PDF) [file pone.0137120.s003.pdf]

S2 Appendix. Sensitivity analyses.

In order to account for the large amount of missing values in the crossed posture condition, we conducted two further analyses to check whether results remain the same when the subjects who performed poorest would be removed from the analyses. In a first analysis, we excluded those subjects for whom more than 2 PSS values had to be excluded. By doing this, we ensured that every participant included in the analyses had at least 2 (out of 4) PSS values remaining in both the uncrossed and the crossed condition. In Experiment 1, 4 participants had to be excluded from the analyses. For the remaining 17 participants, 12 out of 136 (9%) of the values were excluded; all of these were from the crossed posture condition. A chi-squared test indicated that the proportion missing values was significantly larger for the crossed posture (18%) than for the uncrossed posture (0%) (χ^2(1, N = 136) = 11.06; p < 0.001). In Experiment 2, maximum 1 PSS value per participant had to be excluded, so results for Experiment 2 remain the same as reported in section 3.3 and 3.4. For Experiment 1, results of the linear mixed effects model show identical effects as obtained with the original analyses: a main effect of *Laterality* (F(1,106.09) = 22.72; p < 0.001; β = 0.58), and a significant interaction effect between *Laterality* and *Cue Distance* (F(1,104.93) = 13.59; p < 0.001, β = -1.37). Post-hoc analyses show that there was no significant effect of *Cue Distance* in bilateral trials (χ^2(1, N = 17) = 1.34, p = 0.25), however *Cue Distance* had a significant effect in unilateral trials (χ^2(1, N = 17) = 16.75, p < 0.001). The main effect of *Posture* was not significant (F(1,105.47) = 0.26, p = 0.61, β = 0.04), nor was the main effect of *Cue Distance* (F(1,104.41) = 1.28, p = 0.26, β = 0.13). For the JND there were still no significant effects present.

In a second analysis, we excluded all subjects who had on average no 80 percent correct on the trials with the largest SOA (analogous to De Paepe et al. [5]), as this is an indication that participants were not able to perform the task satisfactory. In Experiment 1, 11 participants had to be excluded. For the remaining 10 participants, only three PSS values had to be excluded (4%), and maximum 1 PSS value per participant; all of these were from the crossed posture condition. A Pearson chi square test indicated there was no significant difference in missing values between the uncrossed and the crossed posture condition (

χ^2(N=80) =2.37; p=0.12). In Experiment 2 all participants had on average more than 80% correct, and results remain the same as reported in section 3.3 and 3.4.

For Experiment 1, results of the linear mixed effects model show identical effects as obtained with the original analyses: a main effect of *Laterality* ($F(1,16.25) = 13.32$; $p = 0.002$; $\beta = 0.65$), and a significant interaction effect between *Laterality* and *Cue Distance* ($F(1,54.81) = 5.91$; $p = 0.02$, $\beta = -0.96$). Post-hoc analyses show that there was no significant effect of *Cue Distance* in bilateral trials ($\chi^2(1, N = 10) = 6.42$, $p = 0.79$), however *Cue Distance* had a significant effect in unilateral trials ($\chi^2(1, N = 10) = 89.74$, $p < 0.001$). The main effect of *Posture* was not significant ($F(1,54.79) = 0.02$, $p = 0.88$, $\beta = 0.01$), nor was the main effect of *Cue Distance* ($F(1,54.68)=0.07$, $p = 0.79$, $\beta = -0.03$). For the JND there was a marginally significant main effect of posture ($F(1,8.99) = 3.85$, $p = 0.08$, $\beta = -0.31$), indicating that participants' temporal order judgments were less accurate when their hands were crossed than when their hands were uncrossed. No other significant effects were present ($F < 1.5$, $p > 0.20$).
